# **RETRACTION:** CircRNA EPHB4 modulates stem properties and proliferation of gliomas via sponging miR‐637 and up‐regulating SOX10

**DOI:** 10.1002/1878-0261.13693

**Published:** 2024-07-01

## Abstract

**Retraction:** C. Jin, J. Zhao, Z‐P. Zhang, M. Wu, J. Li, B. Liu, X‐B. Liao, Y‐X. Liao, and J‐P. Liu, “CircRNA EPHB4 modulates stem properties and proliferation of gliomas via sponging miR‐637 and up‐regulating SOX10,” *Molecular Oncology* 15, no. 2 (2020): 596‐622, https://doi.org/10.1002/1878-0261.12830.

The above article, version of record published online on 16 December 2020 in Wiley Online Library (wileyonlinelibrary.com), has been retracted by agreement between the journal Editor‐in‐Chief, Kevin Ryan; FEBS Press; and John Wiley & Sons Ltd. The journal began an investigation following a report from a third party regarding image duplication in Figure 14H between this article and the following articles: Gong et al. [1] and Zhao, et al. [2]. The authors did not respond to requests by the journal and the publisher for original data and an explanation. The retraction has been agreed because the evidence of image duplication across other articles substantially compromises the conclusions of the article. The authors did not respond to our notice of retraction.

References

[1] GongH. , TaoY. , XiaoS. , LiX. , FangK. , WenJ. , HeP. , and MingZ., “LncRNA KIAA0087 suppresses the progression of osteosarcoma by mediating the SOCS1/JAK2/STAT3 signaling pathway,” Experimental & Molecular Medicine 55 (2023): 831‐843, 10.1038/s12276-023-00972-8.37009803 PMC10167219

[2] ZhaoY. , LiC. , ZhangY. , and LiZ. . “CircTMTC1 contributes to nasopharyngeal carcinoma progression through targeting miR‐495‐MET‐eIF4G1 translational regulation axis,” Cell Death & Disease 13, no. 250 (2022), 10.1038/s41419-022-04686-z.PMC893097735301291

**Retraction:** C. Jin, J. Zhao, Z‐P. Zhang, M. Wu, J. Li, B. Liu, X‐B. Liao, Y‐X. Liao, and J‐P. Liu, “CircRNA EPHB4 modulates stem properties and proliferation of gliomas via sponging miR‐637 and up‐regulating SOX10,” *Molecular Oncology* 15, no. 2 (2020): 596‐622, https://doi.org/10.1002/1878-0261.12830.

The above article, version of record published online on 16 December 2020 in Wiley Online Library (wileyonlinelibrary.com), has been retracted by agreement between the journal Editor‐in‐Chief, Kevin Ryan; FEBS Press; and John Wiley & Sons Ltd. The journal began an investigation following a report from a third party regarding image duplication in Figure 14H between this article and the following articles: Gong et al. [1] and Zhao, et al. [2]. The authors did not respond to requests by the journal and the publisher for original data and an explanation. The retraction has been agreed because the evidence of image duplication across other articles substantially compromises the conclusions of the article. The authors did not respond to our notice of retraction.

References

[1] H. Gong , Y. Tao , S. Xiao , X. Li , K. Fang , J. Wen , P. He , and Ming, Z. “LncRNA KIAA0087 suppresses the progression of osteosarcoma by mediating the SOCS1/JAK2/STAT3 signaling pathway,” Experimental & Molecular Medicine 55 (2023): 831‐843, 10.1038/s12276-023-00972-8.37009803 PMC10167219

[2] Y. Zhao , C. Li , Y. Zhang , and Z. Li . “CircTMTC1 contributes to nasopharyngeal carcinoma progression through targeting miR‐495‐MET‐eIF4G1 translational regulation axis,” Cell Death & Disease 13, no. 250 (2022), 10.1038/s41419-022-04686-z.PMC893097735301291

